# Active Tobacco Smoking Impairs Cardiac Systolic Function

**DOI:** 10.1038/s41598-020-63509-3

**Published:** 2020-04-20

**Authors:** Tom Hendriks, Randy van Dijk, Najod A. Alsabaan, Pim van der Harst

**Affiliations:** 1 University of Groningen, University Medical Center Groningen, Department of Cardiology, Groningen, PO box 30.001, 9700 RB The Netherlands; 2 Present Address: Division of Heart and Lungs, Department of Cardiology, University Medical Center Utrecht, Utrecht University, Utrecht, The Netherlands

**Keywords:** Cardiac hypertrophy, Magnetic resonance imaging, Epidemiology

## Abstract

Tobacco smoking is a well-established risk factor for cardiovascular disease, but its direct effect on myocardial structure and function remains unclear. This study investigated the effects of smoking using a nested matched case-control study design. 5,668 participants of the UK Biobank study who underwent cardiovascular magnetic resonance imaging were screened for inclusion. 102 smokers (56 males) with a median age of 56 years were matched to non-smokers based on sex, age, and body surface area. Manual post-processing and feature tracking analyses were performed to determine left ventricular (LV) and right ventricular (RV) structure and function measures. Linear regression analyses were performed to determine the effect of tobacco smoking on imaging measures. Tobacco smoking was associated with increased LV and RV end-systolic volume (4.98 ± 2.08 mL, 5.19 ± 2.62 mL, P = 0.018, 0.049 respectively), reduced LV and RV ejection fraction (β: −2.21 ± 0.82%, −2.06 ± 0.87%, P = 0.007, 0.019 respectively), and reduced absolute measures of LV peak global longitudinal, radial, and circumferential strain (β: 0.86 ± 0.30%, −2.52 ± 0.99%, 1.05 ± 0.32%, P = 0.004, 0.011, 0.001 respectively). Effect sizes were larger in daily smokers compared to occasional smokers. In a general Caucasian population without known clinical cardiovascular disease, active tobacco smoking was dose dependently associated with impaired cardiac systolic function.

## Introduction

Tobacco smoking is associated with an increased incidence of cardiovascular diseases, including myocardial infarction, vascular stroke, peripheral artery disease, and heart failure^1–3^. Pathophysiological effects of tobacco smoking include sympathomimetic effects, reduced oxygen supply to the organs, inflammation, endothelial dysfunction and a pro-thrombotic state^4^.

Large epidemiological studies focusing on the effects of cardiac risk factors on cardiac structure and function indicate an association of tobacco smoking with increased left ventricular (LV) mass and reduced LV systolic function^5–8^. However, other studies show conflicting results^9,10^. Most aforementioned studies were performed in populations with high prevalence of possible confounding factors such as hypertension, diabetes and obesity. Cardiovascular magnetic resonance imaging (CMR) is the gold standard for both structural and functional cardiac assessment, as well as tissue characterization^11^. Advances in imaging post-processing have made it feasible to assess more subtle changes in cardiac contractility using feature tracking analysis to derive measures of myocardial strain^12,13^.

The aim of our study was to determine the effects of tobacco smoking on cardiac structure and function using CMR as the imaging modality of choice.

## Results

### Study population

**Figure 1:**
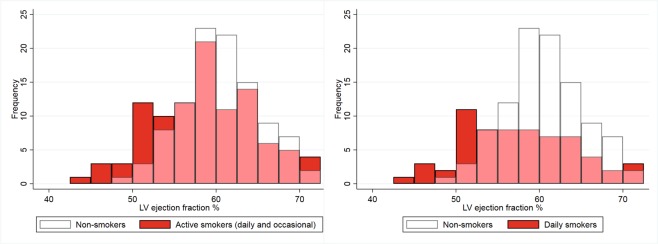
Distribution of left ventricular ejection fraction in smokers vs. non-smokers, and daily smokers vs. non-smokers.

Baseline characteristics are presented in Table 1. In total, 204 participants were included in this study. Mean age of the study population was 58 ± 8 years and 112 subjects (55%) were male. Among the 102 smokers, 64 (63%) were daily smokers and 38 (37%) were occasional smokers. Income category and weekly alcohol units were significantly different between groups. Intraobserver variability in determining imaging measures is presented in Table 2. Smokers had significantly lower mean values of LV ejection fraction (LVEF, 58.6 ± 6.2% vs. 60.6 ± 4.6%, P = 0.011), RVEF (58.3 ± 6.5% vs. 60.5 ± 6.4%, P = 0.019), LV peak global longitudinal strain (−15.6 ± 2.2% vs. −16.2 ± 2.0%, P = 0.024), radial strain (34.8 ± 7.8% vs. 37.1 ± 6.8%, P = 0.024), and circumferential strain (−19.3 ± 2.6% vs. −20.3 ± 2.2%, P = 0.004), compared with non-smokers. Figure 1 demonstrates the difference in distribution of LVEF between smokers and non-smokers, which is most distinct in subjects that smoke on a daily basis.

**Table 1:** Baseline characteristics.

	Non-smokers (N = 102)	Smokers (N = 102)	P
Male sex	56 (54.9%)	56 (54.9%)	1.00
Age, years	56.50 (51.24, 64.48)	56.45 (51.41, 64.05)	0.99
Weight, kg	71.75 (9.92)	72.55 (9.84)	0.56
Height, cm	171.33 (8.67)	170.76 (8.47)	0.64
Body mass index, kg/m^2^	24.39 (2.42)	24.84 (2.49)	0.19
Body surface area, m^2^	1.84 (0.16)	1.84 (0.16)	0.85
Waist hip ratio	0.85 (0.07)	0.86 (0.07)	0.29
Systolic blood pressure, mmHg	128.24 (17.06)	125.85 (14.99)	0.29
Diastolic blood pressure, mmHg	78.56 (8.84)	78.56 (8.08)	1.00
Pulse pressure, mmHg	49.68 (11.53)	47.29 (10.30)	0.12
Heart rate during CMR, bpm	61.60 (10.53)	63.22 (10.24)	0.27
Hypertension	24 (23.5%)	22 (21.6%)	0.74
Antihypertensive medication	13 (12.7%)	9 (8.8%)	0.37
Hyperlipidemia	15 (14.7%)	24 (23.5%)	0.11
Diabetes mellitus	3 (2.9%)	5 (4.9%)	0.47
Chronic obstructive pulmonary disease	23 (22.5%)	18 (17.6%)	0.38
Townsend deprivation index	0.01 (0.94)	0.15 (1.18)	0.37
Average total household income before tax			0.022
<18,000	4 (3.9%)	14 (13.7%)	
18,000–30,999	17 (16.7%)	28 (27.5%)	
31,000–51,999	33 (32.4%)	18 (17.6%)	
52,000–100,000	29 (28.4%)	25 (24.5%)	
>100,000	9 (8.8%)	7 (6.9%)	
Unknown	10 (9.8%)	10 (9.8%)	
Alcohol intake, per week	9.60 (2.26, 15.70)	13.20 (3.90, 24.60)	0.020

Normally distributed continuous variables are presented as mean (standard deviation) and non-normally distributed variables as median (interquartile range). Categorical data are expressed as number (%).

**Table 2:** Intraobserver variability.

	Intraclass correlation coefficient
LV mass	0.96
LV end-diastolic volume	0.99
LV end-systolic volume	0.90
LV ejection fraction	0.77
LV cardiac output	0.93
LV mass to volume ratio	0.88
RV end-diastolic volume	0.99
RV end-systolic volume	0.97
RV ejection fraction	0.85
LV peak global longitudinal strain	0.93
LV peak global radial strain	0.99
LV peak global circumferential strain	0.99

LV, left ventricular; RV, right ventricular.

### Effects of tobacco smoking

Results of the univariate and multivariable regression analyses are presented in Table 3. Active tobacco smoking was a significant predictor of impaired LV and RV systolic function, with regard to EF (β: −2.21 ± 0.82%, −2.06 ± 0.87%, P = 0.007, 0.019 respectively) as well as LV myocardial peak global longitudinal strain (GLS), peak global radial strain (GRS) and peak global circumferential strain (GCS) (β: 0.86 ± 0.30%, −2.52 ± 0.99%, 1.05 ± 0.32%, P = 0.004, 0.011, 0.001 respectively), both in univariate linear regression analyses and after further adjustment for matching parameters and possible confounders. Tobacco smoking was also associated with a higher LV and RV end-systolic volume (ESV) after correcting for multiple confounding variables (β: 4.98 mL, 5.19 mL, P = 0.018, 0.049 respectively). Linear regression analyses were repeated in daily smokers and occasional smokers separately (Table 4). In occasional smokers (N = 38), none of the observed effects of tobacco smoking on imaging biomarkers remained significant (P > 0.05), whereas larger effect sizes and higher significance was observed in the group of daily smokers (N = 64).

**Table 3:** Linear regression results; effects of smoking on measures of cardiac structure and function.

	Univariate			Model 1*			Model 2†		
	Beta	SE	P	Beta	SE	P	Beta	SE	P
LV mass (g)	1.17	2.75	0.67	0.93	1.75	0.60	2.94	1.82	0.11
LV end-diastolic volume (mL)	−0.47	4.66	0.92	−0.74	3.39	0.83	3.26	3.29	0.32
LV end-systolic volume (mL)	3.02	2.58	0.24	2.91	2.00	0.15	4.98	2.08	0.018
LV ejection fraction (%)	−1.97	0.77	0.011	−1.96	0.74	0.009	−2.21	0.82	0.007
LV cardiac output (L/min)	−0.08	0.15	0.60	−0.08	0.13	0.52	−0.08	0.14	0.54
LV mass to volume ratio (g/mL)	0.014	0.012	0.27	0.013	0.011	0.24	0.014	0.011	0.20
RV end-diastolic volume (mL)	0.50	5.39	0.93	0.20	3.88	0.96	3.87	4.09	0.35
RV end-systolic volume (mL)	3.61	3.21	0.26	3.47	2.37	0.15	5.19	2.62	0.049
RV ejection fraction (%)	−2.14	0.90	0.019	−2.13	0.80	0.008	−2.06	0.87	0.019
LV peak global longitudinal strain (%)	0.67	0.29	0.024	0.66	0.29	0.022	0.86	0.30	0.004
LV peak global radial strain (%)	−2.34	1.03	0.023	−2.33	0.90	0.011	−2.52	0.99	0.011
LV peak global circumferential strain (%)	0.98	0.33	0.004	0.98	0.29	0.001	1.05	0.32	0.001

LV, left ventricular; RV, right ventricular.

Reported are unstandardized regression coefficients for active tobacco smoking.

*Corrected for body surface area, age, sex.

^†^Corrected for body surface area, age, sex, diastolic blood pressure, systolic blood pressure, antihypertensive medication, hyperlipidemia, diabetes mellitus, income category, and alcohol intake.

**Table 4:** Linear regression results; comparison of daily with occasional tobacco smoking.

	Occasional tobacco smoking (N = 38)			Daily tobacco smoking (N = 64)		
	Beta	SE	P	Beta	SE	P
LV mass (g)	2.97	2.39	0.22	2.92	2.07	0.16
LV end-diastolic volume (mL)	2.22	4.32	0.61	3.92	3.73	0.30
LV end-systolic volume (mL)	1.30	2.70	0.63	7.28	2.33	0.002
LV ejection fraction (%)	−0.39	1.05	0.71	−3.35	0.91	<0.001
LV cardiac output (L/min)	−0.39	0.18	0.83	−0.11	0.16	0.47
LV mass to volume ratio (g/mL)	0.014	0.015	0.35	0.015	0.013	0.25
RV end-diastolic volume (mL)	5.45	5.39	0.31	2.89	4.65	0.54
RV end-systolic volume (mL)	4.92	3.45	0.16	5.36	2.98	0.073
RV ejection fraction (%)	−1.47	1.14	0.20	−2.43	0.99	0.015
LV peak global longitudinal strain (%)	0.26	0.38	0.50	1.23	0.33	<0.001
LV peak global radial strain (%)	−1.85	1.30	0.15	−2.94	1.12	0.009
LV peak global circumferential strain (%)	0.75	0.41	0.072	1.24	0.36	0.001

LV, left ventricular; RV, right ventricular.

Reported are unstandardized regression coefficients, corrected for body surface area, age, sex, diastolic blood pressure, systolic blood pressure, antihypertensive medication, hyperlipidemia, diabetes mellitus, income category, and alcohol intake.

## Discussion

In this nested matched case-control study, we observed a significant association between active tobacco smoking and impaired systolic LV and RV function, in terms of EF as well as myocardial strain measures. The observed associations were mostly driven by daily tobacco smokers.

Previous studies reporting on the effects of tobacco smoking on cardiac structure and function have shown contradictory results^5–10^. Most studies observed an association between tobacco smoking and reduced systolic function and increased LV mass. The heterogeneity of these study populations possibly influence and obscure the independent effect of tobacco smoking on cardiac structure and function. By using strict in- and exclusion criteria, we selected a relatively healthy population of UK Biobank participants free of major cardiovascular disease and with BMI and blood pressures within normal ranges. We believe that our pre-selection combined with the further correction in our regression models more clearly show the possible independent effects of active tobacco smoking. We were also able to assess more subtle changes in cardiac contractility using strain analysis. We observed that smoking status significantly impaired systolic LV and RV function by increasing ESV, and reducing EF and absolute measures of LV myocardial strain. This effect was mostly driven by daily smokers. Although the study was not designed for this purpose, no significant effect of occasional smoking on cardiac structure and function was found. In our study, tobacco smoking was not associated with increased LV mass, which was observed in several previous studies^5–7,10^. A possible reason could be that LV hypertrophy has a very multifactorial etiology (e.g. metabolic, immunologic, vascular) and we selected our study population to be relatively healthy.

Several large epidemiological studies have suggested tobacco smoking to be a risk factor for impaired cardiac function and heart failure^1,14^, assumed to be driven by myocardial infarctions. Our results seem to indicate that there is a direct relationship between tobacco smoking and subclinical impaired systolic function. Chronic exposure to tobacco smoking has a direct effect on the microvascular compartment, which could be the cause of the observed subtle functional systolic impairment. Tobacco smoke consists of more than 5.000 toxic and carcinogenic chemicals^15^. The (chronic) exposure to these toxic chemicals have potentially devastating effects on cardiac tissue, causing a complex cascade of inflammation, endothelial injury, dysfunction, cell death and fibro-fatty replacement^16^. Biochemically, tobacco smoking has been associated with increased levels of biomarkers for increased wall stress and myocardial injury such as NT-proBNP and high-sensitive troponin T^17^. Former heavy smokers have an increased risk of developing heart failure that former light smokers do not have^18,19^, suggesting permanent myocardial injury related to frequency of smoking or cumulative exposure to tobacco smoke. We studied individuals without known myocardial infarction. However, we cannot rule out that the observed impairment of systolic function is due to the effects of myocardial ischemia resulting from subclinical coronary atherosclerosis, as subjects did not undergo coronary computed tomography or conventional angiography. Further research is needed to assess how tobacco smoking affects diastolic function, and to unravel the mechanisms behind the observed link between tobacco smoking and depressed systolic function.

## Strengths and Limitations

One of the strengths of this study is its design, a nested matched case control study in a relatively healthy population allows for an unbiased interpretation of the effect of tobacco smoking with little confounding. Other strengths include the use of CMR feature tracking analyses. There are some limitations to this study that should be highlighted. This study was a retrospective analysis of participants included in the UK Biobank study. The participants received extensive questionnaires during multiple visits, but there was insufficient information on the extent of tobacco smoke exposure (e.g. pack years) and daily number of cigarettes to include these parameters in the analysis. To address possible confounding we performed regression analysis with correction for confounders known from previous studies. However, there might be residual confounding due to variables other than those included in the analysis, such as a difference in physical activity between smokers and non-smokers, and the observed relationship between tobacco smoking and impaired systolic function should not be interpreted as direct causality.

## Conclusions

In this nested matched case control study within Caucasian subjects of the UK Biobank free of major cardiovascular disease, active tobacco smoking was dose dependently associated with impaired cardiac systolic function as indicated by alterations in end-systolic volume, ejection fraction, and myocardial strain measures. Our findings suggest that smoking cause subclinical changes in ventricular function, even in the absence of evident clinical cardiac disease. Further research is needed to unravel the mechanisms behind the link between tobacco smoking and impaired systolic function.

## Methods

The data for this study is publicly available to registered investigators of the UK Biobank.

### Study population

We performed a nested matched case-control study including 5,668 UK Biobank subjects who underwent CMR assessment. The UK Biobank is a major national health resource including approximately 500,000 participants aged 40–69 years^20^. Written informed consent was obtained in all subjects. UK Biobank’s research ethics committee and Human Tissue Authority research tissue bank approvals mean that researchers wishing to use the resource do not need separate ethics approval. Baseline visits took place between 2006–2010, in which subjects received elaborate touchscreen questionnaires, underwent anthropometric and vital signs measurements, and were interviewed by a trained nurse to determine medical history and medication use. In addition, hospital episode statistics were collected. In 2014, the UK Biobank started with imaging visits, with the aim to perform CMR assessments in 100,000 subjects. During imaging visits, most procedures from baseline visit were repeated.

Baseline characteristics of all participants that were included in the current study were obtained at the imaging visit. Daily smokers were defined as subjects who answered “yes, on most or all days” to the question “do you smoke tobacco now?” during the imaging visit, and occasional smokers were defined as subjects who answered “only occasionally” to the question “do you smoke tobacco now?” and in addition had to have smoked at least 100 times in their lifetime. Body surface area (BSA) was calculated using the equation proposed by Du Bois and Du Bois^21^. Blood pressure was defined as the average of two automated blood pressure measurements. Automated measurements were modified based on the method described by Stang *et al*.^22^. Definitions of chronic obstructive pulmonary disease, hypertension, diabetes mellitus and hypercholesterolemia included medication use. Townsend Deprivation Index (TDI, a proxy for socioeconomic status) was determined at baseline visit by the UK Biobank and inverse rank normalized.

Exclusion criteria were major cardiovascular disease (defined as stroke, transient ischemic attack, cardiac surgery, percutaneous cardiac intervention, pacemaker/implantable cardioverter defibrillator, cardiomyopathy, heart failure, myocardial infarction, peri-/myocarditis, arrhythmia, cardiac arrest, valvular disease, pulmonary arterial hypertension, thromboembolism, coronary artery disease, non-cardiac arterial disease, or atherosclerosis), renal failure, cancer or chemotherapy, former smokers (daily or almost daily) with at least >100 smoked cigarettes in their lifetime, BMI < 16 or >30, systolic blood pressure >160 mmHg or diastolic blood pressure >100 mmHg, and non-Caucasian ethnicity. After applying exclusion criteria, 139 active smokers and 1899 non-smokers were identified. Smokers were matched in a 1:1 ratio with non-smokers based on sex, age (rounded to the nearest year) and BSA (rounded to the nearest decimal). After matching, image quality of CMR assessment was assessed, blinded to study group. If image quality was deemed too poor due to missing short axis stacks, major artifacts or poor axis alignment, smokers were excluded and non-smokers were replaced with a new match, if possible. 14 smokers were not able to be matched with a control and 23 smokers were excluded due to poor image quality, leaving a total of 102 active smokers matched with non-smokers.

### Image acquisition

The UK Biobank CMR protocol has been published previously^23^. In brief, a 20-min CMR protocol was used without administration of contrast agents. A steady-state free precession (SSFP) pulse sequence was used on a 1.5 Tesla scanner (MAGNETOM Aera, Syngo Platform VD13A, Siemens Healthcare, Erlangen, Germany) for the acquisition of a short axis cine stack covering the complete left and right ventricle, and a 2-chamber view (2CV), 3-chamber view (3CV), and 4-chamber view (4CV) long axis cine series.

### Image post processing

Manual post-processing analyses were performed using cvi42 post-processing software (Circle Cardiovascular Imaging Inc., Calgary, Alberta, Canada). LV and RV endocardial and LV epicardial contours were drawn at end-diastolic and end-systolic phases in short axis cine images. LV mass was determined at end diastolic phase, papillary muscles were excluded from LV mass. The cvi42 tissue tracking plugin in beta release 5.3.0.821 was used to determine LV myocardial strain measures. LV endo- and epicardial contours at end diastolic phase in short axis cine images were used to determine peak GRS and peak GCS. To determine peak GLS, LV endo- and epicardial contours were drawn at end-diastolic phase in long axis cine images (2CV, 3CV, 4CV). All post processing analyses were performed by one experienced observer, blinded to study group, according to contemporary consensus recommendations^24^.

### Statistical analysis

Differences in baseline characteristics between smokers and non-smokers were compared using an independent t-test in case of normally distributed continuous variables, a Wilcoxon rank-sum test in case of non-normally distributed continuous variables, and a Pearson’s chi-squared test in case of categorical variables. Intraobserver variability in imaging measures was assessed using intraclass correlation coefficients. The effect size of tobacco smoking on imaging measures was assessed using linear regression analyses. A missing value of alcohol intake per week was imputed with the median value of the study population. Three linear regression models were used; simple univariate linear regression, a multivariable model (*Model 1*) adjusting for the matching parameters (age, sex, and BSA), and a multivariable model (*Model 2*) additionally adjusting for baseline characteristics that were significantly different between groups (income and alcohol consumption) and other cardiovascular risk factors (BMI, diastolic blood pressure, systolic blood pressure, antihypertensive medication, hyperlipidemia, and diabetes mellitus). Multivariable analysis using *Model 2* was repeated using a categorical smoking variable to test independently for the effects of daily tobacco smoking and occasional tobacco smoking. A significance level (α) <0.05 was considered statistically significant. All statistical analyses were performed using Stata/SE version 15.1.
